# Intracardiac Echocardiography Guided Transcatheter Aortic Valve Replacement in a Severe Chronic Obstructive Pulmonary Disease Patient Ineligible for Standard Echocardiography

**DOI:** 10.1002/ccr3.70829

**Published:** 2025-09-02

**Authors:** Hiroki Okamoto, Atsushi Hayashi, Kohei Asada, Yoshihisa Nakagawa

**Affiliations:** ^1^ Department of Internal Medicine, Division of Cardiovascular Medicine Shiga University of Medical Science Otsu Japan

**Keywords:** aortic stenosis, chronic obstructive pulmonary disease (COPD), intracardiac echocardiography, transcatheter aortic valve replacement (TAVR)

## Abstract

Intracardiac echocardiography can be performed under local anesthesia and provides real‐time monitoring with high image quality without interference from the procedure during transcatheter aortic valve replacement, even when transthoracic echocardiography and transesophageal echocardiography guidance are not appropriate owing to the patient's severe chronic obstructive pulmonary disease.

## 1 Introduction

Transcatheter aortic valve replacement (TAVR) has been established as a less invasive option for the treatment of severe aortic stenosis (AS) in patients considered at intermediate or higher perioperative risk [1, 2]. However, TAVR has a risk of complications, including the development of post‐valvuloplasty severe aortic regurgitation (AR) or indication of pericardial effusion suggesting annulus rupture and ventricular perforation, interference of the prosthesis by mitral anterior leaflet or chordae and consequent development of mitral regurgitation (MR), and dislodgment or migration of the transcatheter heart valve (THV) into the aorta or the left ventricle (LV) [3, 4, 5, 6]. These complications cannot be detected at an early stage without echocardiographic guidance [7, 8].

Transesophageal echocardiography (TEE) has long been established as a standard guidance method for intraoperative monitoring during the TAVR procedure. However, its usage has declined in recent years due to the increasing adoption of local anesthesia‐based TAVR techniques, primarily because TEE requires general anesthesia and endotracheal intubation. Centers in Europe and North America have advocated for a “minimalist” TAVR approach whereby patients undergo the procedure under local anesthesia with intermittent transthoracic echocardiography (TTE) guidance. However, some patients cannot be adequately guided using TTE, particularly those with poor images in the supine position because of obesity or lung disease.

Intracardiac echocardiography (ICE) offers the advantage of high image quality using a 9‐Fr sheath inserted via the right jugular vein [9]. Notably, ICE allows for real‐time monitoring without interrupting the procedure [9]. Additionally, ICE can be performed under conscious sedation in patients at high risk of postoperative pulmonary complications following general anesthesia and endotracheal intubation, such as those with severe chronic obstructive pulmonary disease (COPD). Here, we report a case of TAVR guided by ICE necessitated by the unsuitability of TTE and TEE guidance because of severe COPD.

## 2 Case Presentation

### 2.1 Case History and Examination

An 85‐year‐old man with severe AS was referred to our institution for TAVR after presenting with dyspnea on exertion. TTE showed a severely calcified aortic valve (transvalvular peak velocity = 4.0 m/s, mean pressure gradient = 41 mmHg, aortic valve area index = 0.50 cm^2^/m^2^) and normal LV systolic function (ejection fraction = 63%). Thus, he met the criteria for aortic valve replacement due to symptomatic severe AS. He had a mixed obstructive and restrictive ventilatory defect with severe COPD and a history of hospitalization for carbon dioxide narcosis. A pulmonary function test showed a vital capacity of 2.21 L (74% predicted), a forced expiratory volume in the first second (FEV_1_) to forced vital capacity ratio of 30%, and an FEV_1_ of 0.56 L (26% predicted), which was Global Initiative for Chronic Obstructive Lung Disease (GOLD) grade 4. The operative mortality of the patient was estimated at 7.4% using the Society of Thoracic Surgeons risk score. We decided to select TAVR because he was an elderly patient with high surgical risk ineligible for surgical aortic valve replacement due to severe COPD. Electrocardiogram‐gated computed tomography revealed that the aortic valve annulus area was 392 mm^2^ and the circumference was 72.4 mm. Because the iliofemoral artery and aorta were considered adequate for a femoral artery approach, we planned the implantation of a SAPIEN 3 26‐mm valve (Edwards Lifesciences, Irvine, CA, USA) via the transfemoral approach. TEE guidance was not appropriate because general anesthesia was associated with a high risk of postoperative pulmonary complications owing to the patient's severe COPD. We planned to perform TAVR under conscious sedation. However, TTE provided poor images in the supine position. Therefore, we decided to perform TAVR under ICE guidance.

### 2.2 Treatment

**FIGURE 1:**
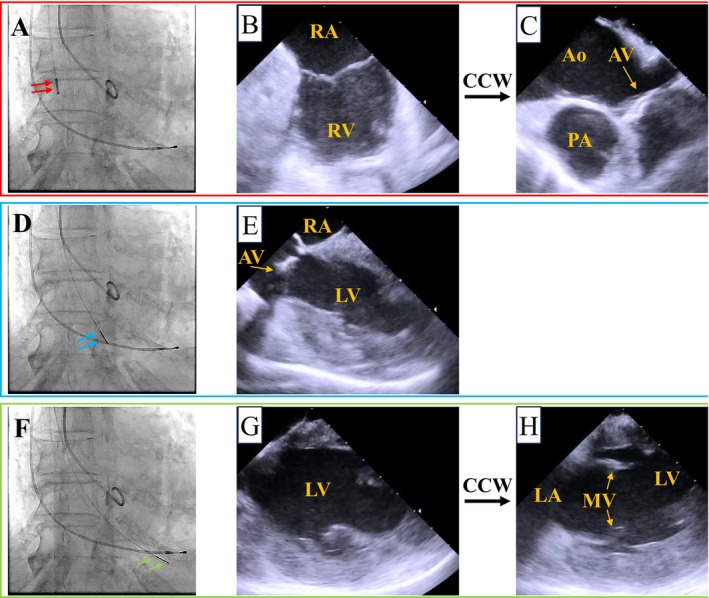
Location of ICE catheter and images obtained at that location. (A) ICE catheter (red arrow) at the right atrium in fluoroscopic view. (B) Long‐axis tricuspid valve view with the catheter at the right atrium. (C) Long‐axis ascending aorta and aortic valve view with the catheter at the right atrium. This view is obtained by counterclockwise rotation from the long‐axis tricuspid valve view. (D) ICE catheter (blue arrow) near the tricuspid valve at the right atrium in fluoroscopic view. (E) Long‐axis left ventricular outflow tract view with the catheter near the tricuspid valve at the right atrium. (F) ICE catheter (green arrow) at the right ventricle in fluoroscopic view. (G) Long‐axis left ventricle view with the catheter at the right ventricle. (H) Long‐axis mitral valve view with the catheter at the right ventricle. This view is obtained by counterclockwise rotation from the long‐axis left ventricle view. Ao, aorta; AV, aortic valve; CCW, counterclockwise; ICE, intracardiac catheter echocardiography; LA, left atrium; LV, left ventricle; MV, mitral valve; PA, pulmonary artery; RA, right atrium; RV, right ventricle.

**FIGURE 2:**
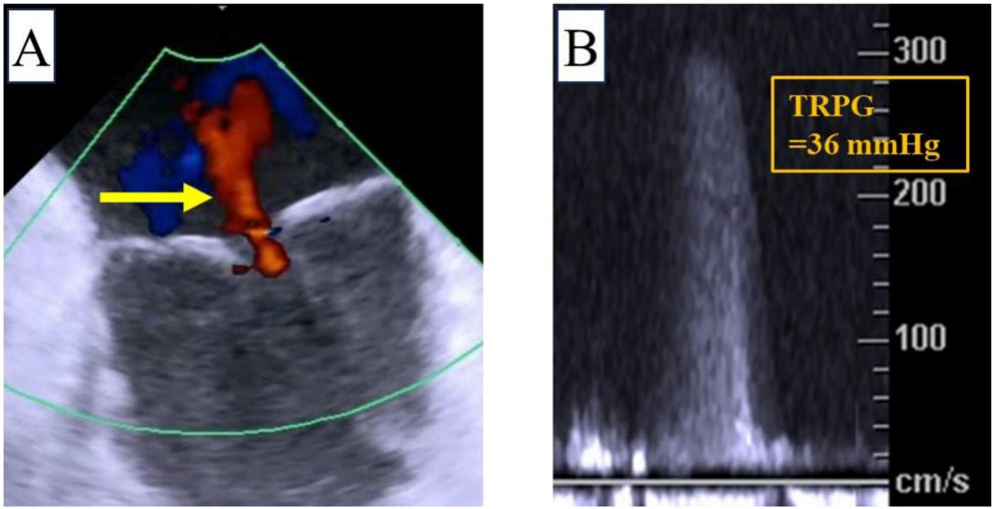
Preoperative assessment of tricuspid valve. (A) Color Doppler flow of tricuspid regurgitation (yellow arrow) in long‐axis tricuspid valve view. (B) Continuous‐wave Doppler across the tricuspid valve. The tricuspid regurgitation pressure gradient was 36 mmHg. TRPG, tricuspid regurgitant pressure gradient.

**FIGURE 3:**
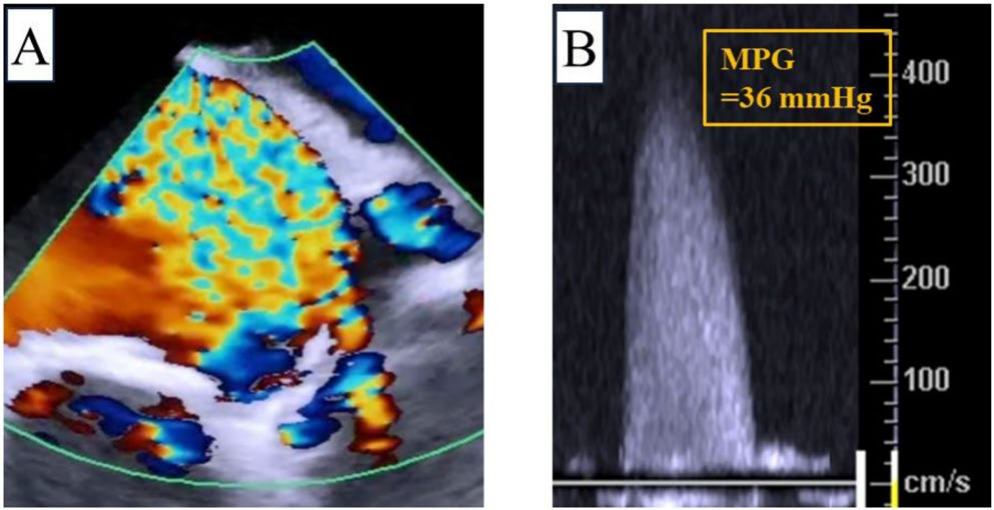
Preoperative assessment of aortic valve. (A) Color Doppler flow in long‐axis aortic valve view. (B) Continuous‐wave Doppler across the aortic valve. The aortic valve mean pressure gradient was 36 mmHg. MPG, mean pressure gradient.

**FIGURE 4:**
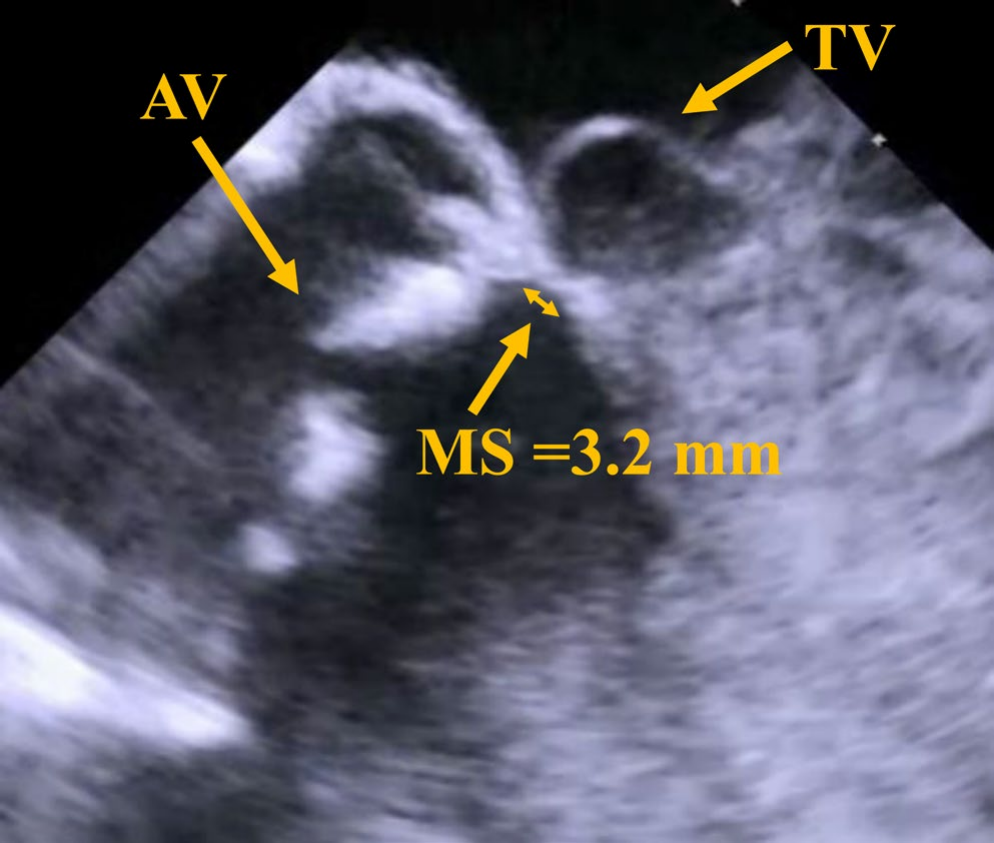
Preoperative assessment of membrane septum. AV, aortic valve; MS, membrane septum; TV, tricuspid valve.

**FIGURE 5:**
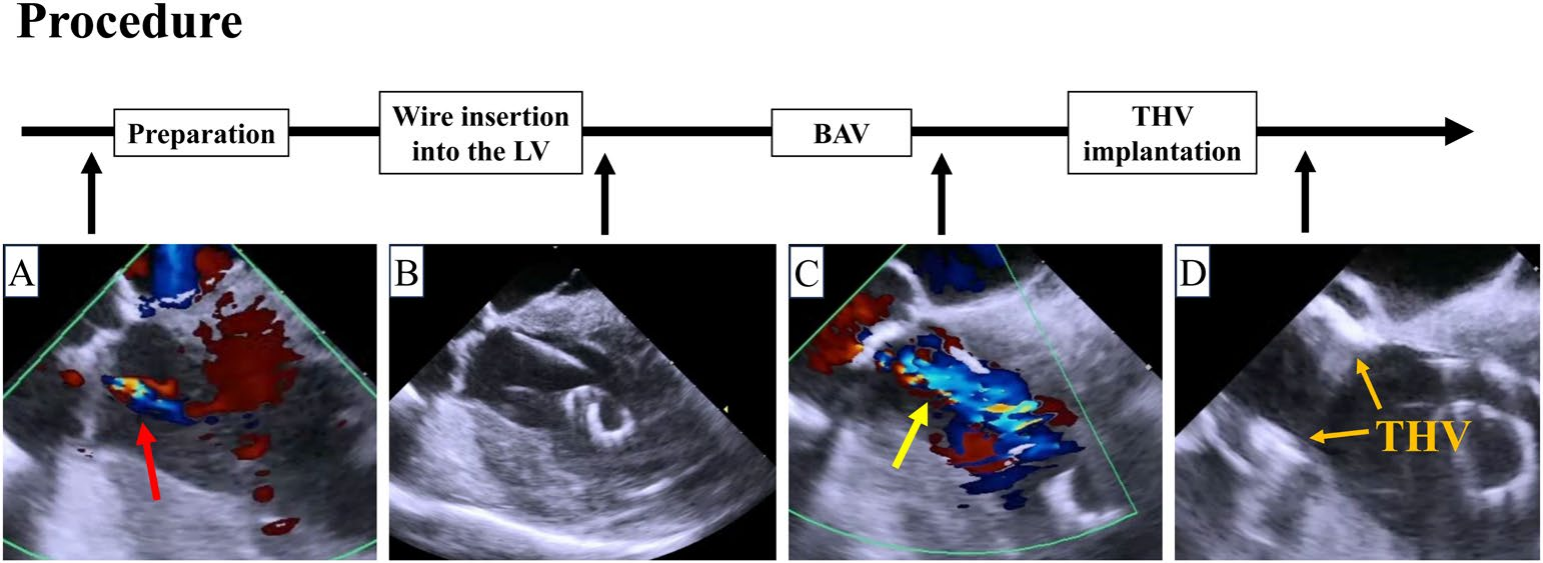
Long‐axis left ventricular outflow tract view during the procedure. (A) The degree of preoperative aortic regurgitation (red arrow) was trivial. (B) Stiff wire was in the optimal position of the left ventricle with the coiled section of the tip at the mid‐apex of the LV and without interference with the mitral valve complex. (C) Aortic regurgitation worsened to moderate after balloon aortic valvuloplasty (yellow arrow). Fortunately, there was no worsening of hemodynamics. (D) Situation after transcatheter heart valve implantation. THV was implanted in the optimal position. BAV, balloon aortic valvuloplasty; LV, left ventricle; THV, transcatheter heart valve.

**FIGURE 6:**
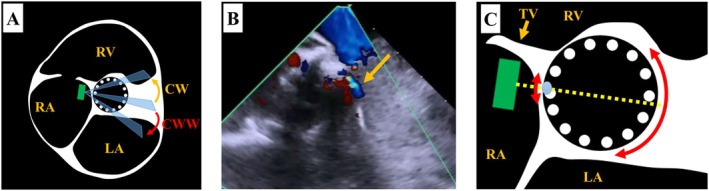
Postoperative assessment of PVL. (A) Illustration of short‐axis view at the level of the aortic valve in transthoracic echocardiography. We assessed PVL by rotating the ICE catheter (green rectangle) at the position near the tricuspid valve in the right atrium. (B) Trivial PVL (orange arrow). (C) Illustration of magnified short‐axis view of aortic valve after transcatheter heart valve implantation. The green rectangle is the ICE catheter. The yellow line is the cross‐section of (B). The light‐blue oval is the location of the PVL. The range of red double arrows is the observable area of PVL in the long‐axis left ventricular outflow tract view with ICE. CW, clockwise; CCW, counterclockwise; ICE, intracardiac catheter echocardiography; LA, left atrium; RA, right atrium; RV, right ventricle; TV, tricuspid valve.

**FIGURE 7:**
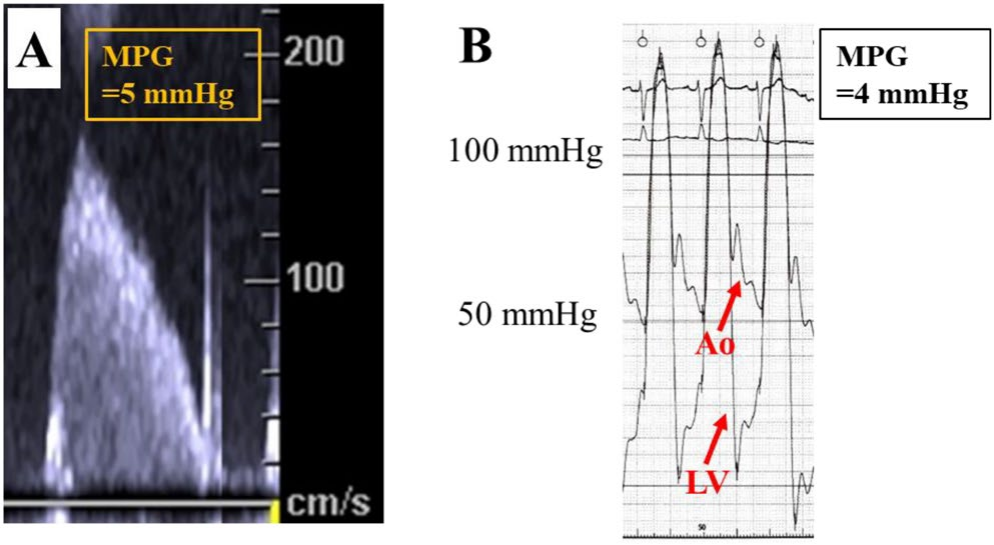
Postoperative assessment of transaortic pressure gradient. (A) Continuous‐wave Doppler across the aortic valve. The mean pressure gradient was 5 mmHg. (B) Pressure waveform after implantation of the transcatheter heart valve. The mean pressure gradient between aorta and left ventricle was 4 mmHg, equivalent to the mean pressure gradient measured by intracardiac echocardiography. Accordingly, the reduction of transaortic pressure gradient by TAVR was acceptable. MPG, mean pressure gradient.

An AcuNav (Biosense Webster, Irvine, CA, USA) ICE catheter was inserted via a 9‐Fr sheath from the right jugular vein. A temporary pacemaker was also inserted via a 6‐Fr sheath from right jugular vein. By pushing the ICE catheter forward approximately 7 cm (Figure 1A), a long‐axis tricuspid valve view was acquired (Figure 1B). Preoperative tricuspid regurgitation was mild, and the tricuspid regurgitant pressure gradient was 36 mmHg (Figure 2A,B). With counterclockwise rotation of approximately 45°, a long‐axis ascending aorta and aortic valve view was acquired (Figures 1C, 3A). The preoperative aortic valve mean pressure gradient was 36 mmHg (Figure 3B). By pushing the ICE catheter slightly for the tricuspid valve with anterior flexion (Figure 1D), a long‐axis LV outflow tract (LVOT) view was acquired (Figure 1E). This view showed LV contraction, pericardial effusion, and AR, and allowed measurement of the membrane septum length, which was 3.2 mm (Figure 4). There was no pericardial effusion in pre‐procedure. Preoperative AR was trivial (Figure 5A). By pushing the ICE catheter into the right ventricle (RV) (Figure 1F), a long‐axis LV view was acquired (Figure 1G), and further counterclockwise rotation of approximately 30° provided a long‐axis mitral valve view (Figure 1H). After Safari guidewire (Boston Scientific, Marlborough, MA, USA) insertion into the LV, we confirmed in the long‐axis LVOT view that the stiff wire was in the optimal position of the LV with the coiled section of the tip at the mid‐apex of the LV and without interference with the mitral valve complex (Figure 5B). Although we confirmed the worsening of AR after balloon aortic valvuloplasty (BAV) (Figure 5C), fortunately there was no worsening of hemodynamics. Thereafter, the THV was implanted during rapid ventricular pacing. After the implantation of the THV (Figure 5D), we performed a scan for any complications on the long‐axis LVOT view. We scanned for paravalvular leakage (PVL) with rotation of the ICE catheter near the tricuspid valve in the long‐axis LVOT view (Figure 6A–C). Trivial PVL from the edge of the non‐coronary cusp was confirmed (Figure 6B). On completion of the procedure, we confirmed no pericardial effusion, and no worsening of LV wall motion and MR. The postprocedural aortic valve mean pressure gradient was 5 mmHg (Figure 7A), equivalent to the mean pressure gradient measured by catheter of 4 mmHg (Figure 7B). Finally, we confirmed no aortic dissection in the long‐axis ascending aorta view. The ICE catheter was removed, and the patient was transferred to the intensive care unit with one in situ sheath and a temporary pacemaker. Immediate postoperative TTE showed no complications.

### 2.3 Outcome and Follow‐Up

Conduction disturbances did not occur during or after the procedure. TTE 5 days after the procedure showed trivial PVL and no pericardial effusion. The subsequent hospitalization was clinically uncomplicated, and the patient was discharged after 7 days. He had an uneventful course without worsening heart failure for several years.

## 3 Discussion

A key advantage of ICE guidance is its capability to provide high‐quality images for most patients, independent of their background. TTE often produces poorer image quality in the supine position compared to the left lateral position. Furthermore, TTE may be challenging or even impossible for some patients, particularly those with obesity or lung disease. On the other hand, although TEE provides high‐quality images, it requires general anesthesia and endotracheal intubation, which are associated with a significant risk of postoperative pulmonary complications, especially in patients with severe COPD [10]. Additionally, TEE is contraindicated in patients with esophageal disease or previous esophageal or gastric surgery. Unlike TTE and TEE, ICE guidance encounters few limitations related to patient background, making it broadly applicable. Yagasaki et al. reported a case in which ICE‐guided TAVR was performed because TEE was unsuitable due to hiatal hernia [11]. In this case, TTE provided poor images in the supine position, and TEE guidance necessitating general anesthesia was unsuitable due to the patient's severe COPD of GOLD 4 grade. Consequently, we opted for ICE guidance for this procedure. ICE provides high‐quality images in all patients without unique settings to enhance image quality. By adjusting depth and highlighting in the same way in TTE and TEE, ICE images are optimized, and their quality is comparable to the figures reported in this report.

A following key advantage of ICE guidance is its continuous display of the aortic valve, LVOT, mitral valve, and LV without any interference from the operator or the fluoroscope during the procedure [9, 11, 12]. Unlike TTE, which interrupts the procedure to obtain images and cannot provide real‐time monitoring, ICE allows for uninterrupted visualization throughout. Although TEE allows real‐time monitoring, the TEE probe interferes with the procedure to obstruct fluoroscopic views especially during THV implantation. This interference makes it difficult to evaluate the aortic valve and the LV simultaneously during the procedure [9, 11]. Therefore, TEE does not fully utilize echocardiography's capabilities for early detection of complications. ICE has potential as an alternative guidance approach for TAVR. The characteristics of each echocardiography are summarized in Table 1.

**TABLE 1:** The characteristics of each echocardiography during TAVR.

	ICE (Transjugular approach)	TEE	TTE
Anesthesia	Conscious sedation and local anesthesia	General anesthesia	Conscious sedation and local anesthesia
Image quality	Consistently good	Consistently good	Occasionally poor
Real‐time monitoring	Consistently available	Available, but the probe could interfere with the procedure to obstruct fluoroscopic views	Unavailable
Assessment of membrane septum	Available	Unavailable	Unavailable
Assessment of PVL	Some areas not assessable due to the assessment by only long axis view	Fully assessable	Fully assessable, but inferior to TEE

Abbreviations: ICE, intracardiac echocardiography; PVL, perivalvular leakage; TAVR, transcatheter aortic valve replacement; TEE, transesophageal echocardiography; TTE, transthoracic echocardiography.

### 3.1 Evaluation of Hemodynamics With ICE

ICE allows for the measurement of transvalvular pressure gradients of the tricuspid and aortic valve coaxially [13, 14]. Therefore, ICE enables continuous monitoring of hemodynamic change throughout the procedure, including before intervention, after BAV, and after deployment of the prosthetic valve. In this case, we measured the aortic valve pressure gradient after THV implantation with ICE, which did not differ from the catheter‐measured aortic valve pressure gradient. Additionally, ICE provides continuous observation of LV contraction, AR, MR, and pericardial effusion during the procedure. This observation allows for the evaluation of these changes simultaneously, aiding in the early detection of disruption in hemodynamics caused by the insertion of wires, BAV, or THV implantation. Notably, we confirmed the worsening of AR following BAV using ICE.

### 3.2 Evaluation of Guidewire Position With ICE

The position of the guidewire is critical for a safe and successful TAVR. If the guidewire becomes entangled in the chordae tendineae or papillary muscles, it can cause acute MR and hemodynamic instability. ICE enables continuous observation of the wiring in the LV. Therefore, if the guidewire position is not appropriate, we can promptly alert the operator. In contrast, during TAVR guided by TTE, the operators must pause their activity, switch from the guidewire to the echocardiography probe, and assess the guidewire position. This process is not only time‐consuming but may also precipitate hemodynamic collapse. Moreover, TTE does not always provide a clear image of the guidewire in the LV, leading to a further delay in evaluating the cause of hemodynamic instability. This delay can exacerbate hemodynamic deterioration, creating a vicious cycle. Similarly, under TEE guidance, the TEE probe may obscure the fluoroscopic view when evaluating the guidewire in the LV, preventing continuous monitoring. Consequently, TEE, like TTE, does not enable rapid assessment of the guidewire position in comparison to ICE.

### 3.3 Evaluation of PVL With ICE

We consider that ICE may not be the most suitable method for evaluating PVL in comparison with TTE or TEE because obtaining a short‐axis view of the aortic valve with ICE can be challenging. Although forcefully flexing the ICE catheter anteriorly in the RV can provide a short‐axis view of the aortic valve, this procedure requires extensive training under fluoroscopic guidance and may carry risks of complications such as perforation of the RV and ventricular arrhythmias. Therefore, we usually assess PVL only in the long‐axis LVOT view with ICE. We rotate the ICE catheter and evaluate PVL as illustrated in Figure 6A. This method enables us to detect PVL located at the approximately 2–5‐o'clock and 8–10‐o'clock positions in the short‐axis view of the aortic valve obtained with TTE as shown in Figure 6C. Therefore, it is important to note that using this approach may result in missing PVL from other locations.

### 3.4 Evaluation of Membrane Septum With ICE

Several studies have reported that the length of the membrane septum independently predicts the incidence of conduction disturbances following TAVR [15, 16]. Unlike TTE and TEE, ICE provides clear images of the membrane septum and allows for precise measurement of its length. ICE guidance ensures continuous visualization of the membrane septum during the deployment of self‐expandable THVs, minimizing the risk of the distal edge of the THV encroaching on the muscular septum [17, 18, 19, 20]. Consequently, ICE guidance has the potential to decrease the incidence of conduction disturbances associated with TAVR. Additionally, this approach may reduce radiation exposure for both patients and catheter operators.

### 3.5 Limitations of ICE

ICE has several limitations. First, ICE guidance requires insertion of an additional venous sheath. Second, the ICE catheter may injure the heart and vessels and induce arrhythmias. Third, the ICE catheter may interfere with the pacemaker lead. Fourth, the use of ICE increases costs. Fifth, ICE offers a limited view compared to TEE and TTE. This limitation is due to the restricted maneuverability of the ICE catheter and its confined imaging window, limited to the superior vena cava, right atrium, and right ventricle. However, the advent of 3‐dimensional ICE is expected to resolve the issue [21]. Sixth, training is required to operate the ICE catheter and interpret the images. Seventh, operators of the ICE catheter are exposed to scattered radiation originating from beneath the patient bed [22]. Therefore, proper education and the use of adequate shielding to minimize radiation exposure during the procedure are essential. Although ICE has these limitations, it has potential as an alternative guidance for TAVR because ICE guidance may minimize the complications of TAVR [23].

In conclusion, ICE consistently delivers high‐quality images and real‐time monitoring without interfering with the procedure, making it a valuable option for TAVR when TTE or TEE guidance is not appropriate.

## Author Contributions

**Hiroki Okamoto:** conceptualization, resources, visualization, writing – original draft. **Atsushi Hayashi:** writing – review and editing. **Kohei Asada:** resources. **Yoshihisa Nakagawa:** supervision.

## Consent

Written informed consent was obtained from the patient for publication of this case report and any accompanying images.

## Conflicts of Interest

The authors declare no conflicts of interest.

## Data Availability

The data that support the findings of this study are available from the corresponding author upon reasonable request.
